# Clinical Characteristics of Cutaneous Melanoma and Second Primary Malignancies in a Dutch Hospital-Based Cohort of Cutaneous Melanoma Patients

**DOI:** 10.1155/2009/479183

**Published:** 2009-12-31

**Authors:** Haike M. J. van der Velden, Michelle M. van Rossum, Willeke A. M. Blokx, Jan B. M. Boezeman, Marie-Jeanne P. Gerritsen

**Affiliations:** ^1^Department of Dermatology, Radboud University Nijmegen Medical Centre, P.O. Box 9101, 6500 HB Nijmegen, The Netherlands; ^2^Department of Pathology, Radboud University Nijmegen Medical Centre, P.O. Box 9101, 6500 HB Nijmegen, The Netherlands

## Abstract

The increasing number of living cutaneous melanoma patients and the increased risk of developing a second primary tumour incited us to analyse the clinical characteristics of cutaneous melanoma and define the frequency, site, and type of second primary cancers in cutaneous melanoma patients. We collected data on patients who visited the Department of Dermatology at the Radboud University Nijmegen Medical Centre and were newly diagnosed with cutaneous melanoma or metastasis of melanoma with unknown primary localization between 2002 and 2006. A total of 194 cases were included; eleven patients developed a subsequent melanoma, 24 had at least one basal cell carcinoma, three had at least one squamous cell carcinoma, and 21 patients had a second non-cutaneous primary malignancy. In conclusion, 48 patients developed a subsequent malignancy. As nonmelanoma skin cancer is the most frequent second malignancy, our results subscribe to the necessity of follow-up by a dermatologist.

## 1. Introduction

A significant increase in the incidence of cutaneous melanoma (CM) has been observed worldwide, leading to a growing demand for healthcare services [1–9]. In 2003, melanoma accounted for 2869 cases of cancer in The Netherlands. Unfortunately, incidence rates are expected to keep rising in the future. The number of new cases in The Netherlands is expected to be more than 4800 in 2015 [1]. Although absolute mortality rates of CM increased over the last 40 years [2, 9], long-term survival rates are improving [4, 7, 10], mainly due to earlier detection and improving awareness of patients on skin changes [7, 11]. Thus, the total number of living CM patients is increasing, fixing one's attention to detection of metastasis, recurrence, and long-term complications, such as second tumours.

Patients with CM have an increased risk of developing a second primary melanoma [12–18]. Several studies also showed a higher incidence of subsequent nonmelanoma skin cancers (NMSCs), comprising basal cell carcinoma (BCC) and squamous cell carcinoma (SCC), in patients with CM in comparison with the general population [14, 17–21].

There is also a significantly higher risk of a wide variety of primary noncutaneous malignancies among patients with CM [12, 14, 16–19, 21–26].

The increasing number of living CM patients and the increased risk of developing a second primary tumour incited us to analyse the clinical characteristics of cutaneous melanoma and to define the frequency, site, and type of second primary cancers in our patients.

## 2. Material and Methods

### 2.1. Design

We performed a historical cohort study among patients with cutaneous melanoma at the Department of Dermatology of the Radboud University Nijmegen (RUN) Medical Centre (MC), The Netherlands.

### 2.2. Collecting Data

We selected patients with histologically confirmed first CM or metastasis of melanoma with unknown primary localization, diagnosed in the period between January 2002 and December 2006. These patients were included when visiting the Department of Dermatology.

Clinical data were abstracted from the patients' dermatology hospital charts and the Comprehensive Cancer Centre East (IKO), covering the eastern part of The Netherlands. Information on the patient was obtained from the electronic patient database of our hospital up to the first of June 2007.

Data recorded from each patient included sociodemographic and phenotypic characteristics. Furthermore, we gathered information on family history and the occurrence of Dysplastic Nevus Syndrome (DNS). We distinguished two types of DNS. A familial type DNS was defined as cases of a positive family history (i.e., two or more first-degree relatives or three or more second-degree relatives who suffered from melanoma). Individuals with melanoma, five or more clinically atypical nevi, and a negative family history of melanoma (first- and second-degree relatives) were defined as sporadic type DNS [27].

Cutaneous and noncutaneous malignancies occurring either before or after the diagnosis of CM were recorded. The patients were considered to have a second primary cancer when they reported it themselves or when the IKO registered it. Contrary to the rules of the International Agency for Research on Cancer/International Association of Cancer Registries (IARC/IACR) [28] multiple CM arising in the same patient were included in our definition of a multiple primary malignancy. On the other hand, we did exclude recurrences of the melanoma.

### 2.3. Statistical Analysis

Statistical analysis was performed using the Statistical Package for Social Sciences (SPSS) for Windows (release 14.0.2). Throughout the analysis, *P* values <.05 were considered statistically significant. For descriptive purposes, valid percentages (percentages based on sample size excluding missing values) were calculated. An actuarial survival curve was generated using the Kaplan-Meier method for censored data.

## 3. Results

### 3.1. Clinical Data

**Figure 1:**
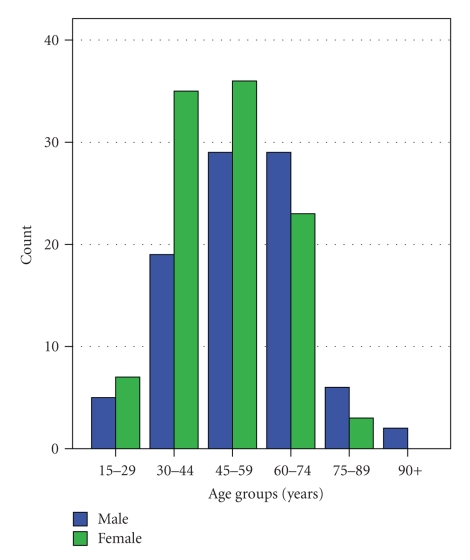
Age of the patients at the time of first melanoma diagnosis.

We studied 194 cases, 90 males (46.4%) and 104 females (53.6%); the difference between both frequencies was not significant (*P* = .351 based on Z approximation). The average age of the patients at diagnosis of the first CM was 51.8 years (SD 15.4) (Figure 1). We registered 158 invasive CM, 21 in situ CM and 15 metastases with unknown primary melanoma. The tumours were predominantly located on the trunk, in men (42.4%) and in women (38.5%) (Table 1). Reported symptoms on the lesion were itch (*n* = 23), pain or discomfort (*n* = 8), spontaneous bleeding (*n* = 23), and change of appearance (*n* = 82). For 79 to 88 subjects, varying among these variables, this information was missing. Superficial spreading melanoma (SSM) was the most frequent histological type (63.9%), followed by nonspecified melanoma (8.8%) and nodular melanoma (NM) in 8.2% of the patients. Lentigo maligna melanoma (LMM), acral-lentiginous melanoma (ALM), amelanotic malignant melanoma (AMM), and other subtypes were less frequently observed (Table 1).

In Table 1 tumour thickness and invasiveness of the primary CM are described. Median and mean tumour thicknesses according to Breslow [29] were 0.75 mm and 1.38 mm (SD 2.23), respectively. Clark invasiveness [30] was available in 87.2% of the first primary CM.

Twenty-five melanomas were associated with a dysplastic nevus and 10 with a nevus naevocellularis. The tumour was ulcerative in 21 patients and the same number showed partial or complete regression. In just a few cases (*n* = 2) the pathologist found satellites in the first pathological examination.

We were able to determine the TNM stage (AJCC) [31, 32] for 172 (88.7%) first CM: 113 (65.7%) stage I, 8 (4.7%) stage II, 26 (15.1%) stage III, and 25 (14.5%) stage IV.

In 179 patients the localization of the primary CM was known: they all underwent excision, and 171 were followed by reexcision. Lymph nodes were investigated in 71 subjects by means of lymph node puncture, lymph node dissection, and/or a sentinel node procedure. In 44 patients, CM had metastasized to the lymph nodes (i.e., positive), with a maximum count of 20 positive nodes per patient. Twenty-nine patients participated in a study, which comprised research on dendritic cell (DC) vaccinations (*n* = 18), observational arm of the DC vaccination study (*n* = 4), a study with Ganglioside (*n* = 4), a PEG interferon study (*n* = 2), and a study on Ticilimumab (*n* = 1). Fifteen patients in this cohort underwent chemotherapy and 12 palliative radiotherapy as treatment of their melanoma.

We tried to document and analyse the follow-up of the patients but failed because each patient seems to have another follow-up scheme. Some of them asked for a higher frequency of controls, while others repeatedly did not show up. Furthermore, various patients had more than one reason to consult the dermatologist.

### 3.2. Risk Factors

There are multiple risk factors for melanoma. We tried to evaluate country of origin, phenotype, social class, medication, ultraviolet radiation (UVR) exposure, immune status, previous radiotherapy and/or chemotherapy, and life style factors, such as diet and alcohol use. Information about most of these factors was incomplete, because this was not routinely collected. Only the variables with enough data are mentioned.

According to the dermatologists 26.8% of the patients had an exceptionally high count of nevi and 10.3% an exceptionally low count. 6.7% had radiotherapy in the past and 1.5% chemotherapy. The number of immune-compromised patients was small (1.5%). Eight (4.1%) patients reported use of any kind of immunosuppressive medication. 

Family history of melanoma was positive in 24 patients (valid percentage 14.6), negative in 140 (valid percentage 85.4), and unknown in 30. There was no significant difference between a positive and negative family history of melanoma with regard to the occurrence of BCC or noncutaneous cancer in this study group. Eight patients cited the occurrence of intestinal cancer in first- and second-degree relatives, seven patients named lung cancer, and two cancer of the prostate. Eighteen patients mentioned relatives with breast cancer, three ovarian cancer, and three had relatives with cervical cancer. Two patients mentioned the occurrence of pancreas carcinoma in third-degree relatives. Other cancers among relatives were liver cancer, renal cell cancer, bladder cancer, testis cancer, Hodgkin lymphoma, leukaemia, and undefined cancers. Two subjects complied with the definition of familial type DNS and four had sporadic type DNS. Three of them requested for genetic examination, but none of them had the particular gene mutation.

### 3.3. Second Primary Tumours

Eleven patients (6.1%) with a first primary melanoma, four males and seven females, developed a second CM. The average age of the patients at diagnosis of the subsequent CM was 47.6 years (SD 13.9). In one individual the first and the second CM were simultaneously diagnosed, one at the thorax and the other at the forearm. The median time interval between the diagnoses was 13.4 months (range 0–29.2 months). In four patients the subsequent CM developed within the same anatomic place, whereas in seven patients they developed in different anatomic places. Median and mean tumour thicknesses according to Breslow were 0.40 mm and 0.49 mm (SD 0.66), respectively. Table 2 describes the characteristics of the second CM.

**Figure 2:**
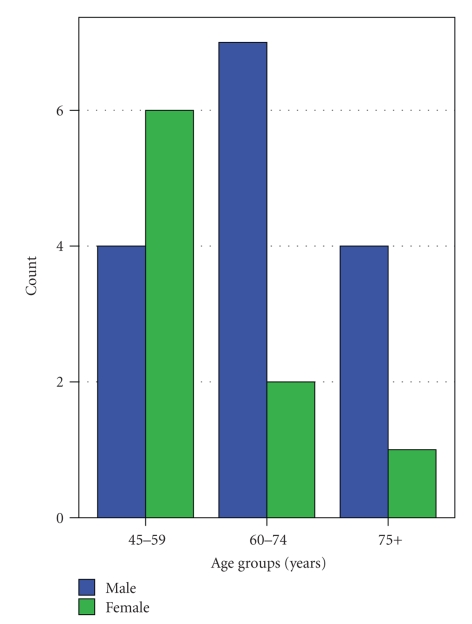
Age of the patients at the time of first basal cell carcinoma diagnosis.

In our group of patients 12.4% had one or more basal cell carcinoma (BCC). Male-female ratio was 15 : 9. We documented a total of 83 BCCs: 11 patients developed one BCC, 3 patients developed two, 2 patients three, and 8 patients four or more BCCs. 68.7% of the BCCs occurred before the first CM, 12.0% simultaneously, and 19.3% after the first CM. Mean age at the diagnosis of the first registered BCC was 64.5 years (SD 11.6) (Figure 2). With respect to histological type, the number of cases was distributed as follows: superficial 39.8%, nodular 25.3%, infiltrative 4.8%, morphea type 1.2%, and unknown type 9.6%. The remaining 19.3% had a combination of different histological types. The tumours were most frequently located at the trunk (42.2%) and in the head/neck region (43.4%). Three male patients (1.5%) were diagnosed with squamous cell carcinoma (SCC). Two patients developed two SCCs and the other only one. In two patients their first SCC preceded the first CM and in one it occurred after the CM.

Twenty-six primary noncutaneous cancers were identified in 21 patients (10.8%) (Table 3). Seventeen patients had noncutaneous cancer diagnosed before CM, three patients had subsequent cancer after the diagnosis of melanoma, and date of diagnosis was missing in one patient. Three of the patients had both melanoma and noncutaneous cancer diagnosed in the same year.

**Figure 3:**
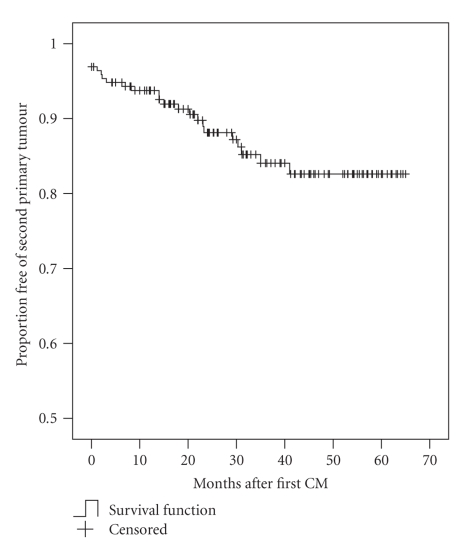
Actuarial survival curve representing the probability of a second primary tumour (cutaneous and noncutaneous) in monthly intervals in the study population.

We calculated the probability for a subsequent primary tumour (a second primary CM, NMSC and/or noncutaneous cancer) by means of the Kaplan-Meier method. Figure 3 shows the actuarial survival curve representing the probability of a second primary tumour after the first CM. Data of patients without the event were censored at the date of the last check of the electronic patient database (1st June 2007) or at their time of death.

## 4. Discussion

We collected data on 194 patients suffering from CM in order to analyse the clinical characteristics of CM and to define the frequency, site, and type of second primary cancers in CM patients.

The distribution of sex and age of patients with CM corresponded well with European data [4, 7, 8, 10, 11, 33]. According to literature, anatomical distribution of melanoma is sex dependent. The most common areas are the trunk for men and the arms and legs for women [4, 5, 8, 10, 11, 33, 34]. This dissimilarity between the sexes could be explained by differences in sun exposure patterns, which matches differences in usual clothing patterns and hair cover [35]. Nevertheless, a recent study stated that in 2004 the only difference in site distribution between both sexes was an increased proportion of head and neck melanomas in men. Most common area in both, males and females, was the trunk [36]. In our cohort most CM were located on the trunk, both in men (42.4%) and women (38.5%), followed by the distal extremities (21.1% in men; 33.7% in women).

Distribution of histological type of melanoma, median tumour thickness according to Breslow, and documented Clark invasiveness corresponded well with previous studies [4, 8, 10, 11, 33]. Twenty-six percent of the primary melanomas reviewed by Bevona et al. were histologically associated with nevi [37]. We found a percentage of 19.6%. Our findings with regard to ulceration and regression corresponded with that of the Central Malignant Melanoma Registry of the German Dermatological Society [10].

A positive family history of melanoma is considered to be a strong risk factor for the occurrence of melanoma. The risk of developing a CM is approximately 2 times higher in persons with a history of melanoma in a first-degree relative compared to the risk of those without [38–40]. In a study conducted by Tucker et al., the excess risk of CM in the melanoma-prone families remained restricted to those participants with prior melanoma or dysplastic nevi [41]. According to literature approximately 10% of the melanoma patients have a positive family history [42]. In our study 14.6% had a positive history. This difference can be explained by the relatively high count of missing values (15.5%). A family history of noncutaneous cancers could be a risk factor for melanoma and be a part of a hereditary cancer syndrome. Bergman et al. observed a significant excess of gastrointestinal cancer in nine FAMMM (familial atypical multiple mole syndrome) families in The Netherlands [43]. More recent, familial syndromes of melanoma and tumours of the nervous system [44], breast cancer [45], and/or pancreatic cancer [45–49] have been documented. Furthermore, melanoma clusters in families with familial retinoblastoma, Li-Fraumeni cancer syndrome, and Lynch syndrome type II [42, 50, 51]. In our study the most frequent cancer among relatives was, as expected on the basis of above mentioned literature, breast cancer, followed by intestinal cancer and lung cancer. Just two patients reported on pancreas carcinoma in third-degree relatives.

Among the 179 primary CM studied, 11 patients (6.1%) developed a second CM. Previous studies found incidences ranging from 2.0% to 8.0% [13, 15, 16, 52–54]. In accordance with previous studies, our data indicate that most subsequent CM occur within the first years after the initial diagnosis [13, 17, 53, 54]. The latter subscribes the importance of a follow-up after the first melanoma diagnosis. The subsequent CM developed in 36.4% of the patients at the same location as before. Percentages found in other studies varied from 34.1% to 51% [53–55]. SSM was the most frequently found histological subtype in both the first and second CM. This is consistent with a previous study [52], although Savoia et al. indicated a higher risk of second CM in patients with the LMM type [54]. We observed a much higher incidence of BCC (12.4% with in total 83 BCC) than SCC (1.5% with in total 5 SCC) in our study cohort. Other studies conducted in a cohort of cutaneous melanoma patients found a ratio BCC:SCC between 7 : 1 and 9 : 1 [19, 20, 52]. The ratio in the overall population varies between 3 : 1 and 8 : 1 [6, 33, 56]. Our results regarding sex and age distribution correspond well with earlier reports [6, 14, 20, 33, 52, 56]. Most common histological subtypes were superficial and nodular BCC, which is consistent with another Dutch study [57]. It is known that BCC typically arise in sun-exposed regions, like the head and neck [20, 33, 52, 56, 57]. In this cohort tumours were most frequently located at the trunk, followed by the head/neck region. The correlation between NMSC and melanoma risk could be the effect of shared risk factors, like phenotype and UVR exposure [58–61]. Because of the retrospective nature of this study and the fact that the information is not routinely collected, such a hypothesis cannot be tested. Furthermore, a genetic susceptibility for skin cancers could explain the coexistence of different types of skin cancers in the same patient.

The current study is hospital based. For that reason, it cannot be automatically extrapolated to the general population. However, the clinical data for melanoma are closely comparable to the existing literature and may thus be regarded as representative. The considerable number of missing data is an important limitation in our study, especially with regard to risk factors. The strength of our study is the combined use of data from the cancer registries, patients' hospital chart and the electronic patient database, which implies that our data were more detailed than data from the general cancer registration.

Two possible selection biases should be taken into account. First, there is an increased surveillance by the patient and by physician after first diagnosis of cancer. This may lead to early and increased detection of subsequent malignancies. Second, differences in the lifestyle (e.g., sunbathing habit) of the population in our study and the general population cannot be excluded and may have influenced the results. In addition, most published articles analysed the occurrence of a second primary malignancy subsequent to melanoma. We investigated the cutaneous and noncutaneous malignancies occurring either before or after the diagnosis of CM.

Finally, statistical analyses of the risk of a second primary tumour in CM patients could not be performed as a consequence of the relatively small number of patients registered. We recommend to continue this registry to attain a larger cohort and achieve more statistical power. Furthermore, the use of a standard oncologic questionnaire by the physician might improve the quality and quantity of information on risk factors for CM.

In conclusion, in the present study 48 CM patients (24.7%) developed a subsequent malignancy. Therefore it seems feasible to monitor CM patients for signs of subsequent malignancies. As NMSC is the most frequent second tumour, our results subscribe to the need of follow-up by a dermatologist.

## Figures and Tables

**Table 1:** Descriptive statistics for the first cutaneous melanoma (CM 1).

	Males *n* (%)	Females *n* (%)	Total *n* (%)
Tumour invasiveness			
In situ	6 (6.7)	15 (14.4)	21 (10.8)
Invasive	75 (83.3)	83 (79.8)	158 (81.4)
Metastasis^a^	9 (10.0)	6 (5.8)	15 (7.7)
Localization			
Head/neck	14 (15.6)	12 (11.5)	26 (13.4)
Trunk	38 (42.4)	40 (38.5)	78 (40.2)
Proximal extremities	10 (11.1)	11 (10.6)	21 (10.8)
Distal extremities	19 (21.1)	35 (33.7)	54 (27.8)
Unknown primary	9 (10.0)	6 (5.8)	15 (7.7)
Histological type			
SSM^b^	54 (60.0)	70 (67.3)	124 (63.9)
NM^c^	10 (11.1)	6 (5.8)	16 (8.2)
LMM^d^	5 (5.6)	8 (7.7)	13 (6.7)
ALM^e^	3 (3.3)	1 (1.0)	4 (2.1)
AMM^f^	1 (1.1)	1 (1.0)	2 (1.0)
Spitzoid melanoma	1 (1.1)	0	1 (0.5)
STUMP^g^	1 (1.1)	1 (1.0)	2 (1.0)
Melanoma, not specified	6 (6.7)	11 (10.6)	17 (8.8)
Unknown primary	9 (10.0)	6 (5.8)	15 (7.7)
Breslow tumour thickness (mm)			
<1.01	44 (54.3)	70 (71.4)	114 (63.7)
1.01–2.00	17 (21.0)	18 (18.4)	35 (19.6)
2.01–4.00	11 (13.6)	7 (7.1)	18 (10.1)
>4.00	9 (11.1)	3 (3.1)	12 (6.7)
Clark invasiveness			
I	6 (8.5)	15 (17.6)	21 (13.5)
II	20 (28.2)	25 (29.4)	45 (28.8)
III	19 (26.8)	21 (24.7)	40 (25.6)
IV	23 (32.4)	21 (24.7)	44 (28.2)
V	3 (4.2)	3 (3.5)	6 (3.8)

^a^Unknown primary melanoma; ^ b^superficial spreading melanoma; ^c^nodular melanoma; ^d^lentigo maligna melanoma; ^e^acral-lentiginous melanoma; ^f^amelanotic malignant melanoma; ^g^spitzoid tumour of unknown malignant potency.

**Table 2:** Descriptive statistics for the second cutaneous melanoma (CM 2).

	Males *n* (%)	Females *n* (%)	Total *n* (%)
Tumour invasiveness			
In situ	1 (25.0)	4 (57.1)	5 (45.5)
Invasive	3 (75.0)	3 (42.9)	6 (54.5)
Localization			
Head/neck	0	0	0
Trunk	2 (50.0)	3 (42.9)	5 (45.5)
Proximal extremities	1 (25.0)	2 (28.6)	3 (27.3)
Distal extremities	1 (25.0)	2 (28.6)	3 (27.3)
Histological type			
SSM^a^	3 (75.0)	3 (42.9)	6 (54.5)
LMM^b^	0	1 (14.3)	1 (9.1)
Melanoma, not specified	1 (25.0)	3 (42.9)	4 (36.4)
Breslow tumour thickness (mm)			
<1.01	3 (75.0)	7 (100)	10 (90.9)
1.01–2.00	0	0	0
2.01–4.00	1 (25.0)	0	1 (9.1)
>4.00	0	0	0
Clark invasiveness			
I	1 (33.3)	4 (57.1)	5 (50.0)
II	0	1 (14.3)	1 (10.0)
III	2 (66.7)	2 (28.6)	4 (40.0)
IV	0	0	0
V	0	0	0

^a^Superficial spreading melanoma; ^b^lentigo maligna melanoma.

**Table 3:** Observed number of cases of noncutaneous malignancies in CM patients.

Site	Observed number		
	Both sexes	Males	Females
Tonsil	1	1	0
Plasmacytoma	2	2	0
Leukaemia	1	1	0
Pancreas	1	0	1
Kidney	2	2	0
Bladder	2	2	0
Lung	3^a^	3^a^	0
Breast	7^a^	2^a^	5
Cervix	2^b^	—	2^b^
Prostate	5	5	—

^a^One tumour in situ, ^b^two tumours in situ.
